# Integrated Analysis of Microarray, Small RNA, and Degradome Datasets Uncovers the Role of MicroRNAs in Temperature-Sensitive Genic Male Sterility in Wheat

**DOI:** 10.3390/ijms23158057

**Published:** 2022-07-22

**Authors:** Yongjie Liu, Dan Li, Shengquan Zhang, Liping Zhang, Jie Gong, Yanhong Li, Jiamin Chen, Fengting Zhang, Xiangzheng Liao, Zhaobo Chen, Yongbo Wang, Binshuang Pang, Jinxiu Ma, Xianchao Chen, Jiangang Gao, Changping Zhao, Shiqing Gao

**Affiliations:** 1 Institute of Hybrid Wheat, Beijing Academy of Agriculture and Forestry Sciences, Beijing 100097, China; liu_yongj@126.com (Y.L.); qdlidan@126.com (D.L.); zsq8200@126.com (S.Z.); lpzhang8@126.com (L.Z.); gjrice@163.com (J.G.); liyanhong201217@163.com (Y.L.); binglinghua66@163.com (J.C.); lyezh@163.com (F.Z.); xiangzhengliao@163.com (X.L.); chzhaobo@126.com (Z.C.); sky999@126.com (Y.W.); pangbinshuang1122@aliyun.com (B.P.); jinxiuma@163.com (J.M.); xcchen10@sina.com (X.C.); gjg86520@126.com (J.G.); 2 Molecular Genetic Beijing Key Laboratory of Hybrid Wheat, Beijing 100097, China; 3 College of Horticulture, China Agricultural University, Beijing 100193, China

**Keywords:** *Triticum aestivum* L., temperature-sensitive genic male sterility (TGMS), miRNA, cell plate, auxin, lipid metabolism, degradome

## Abstract

Temperature-sensitive genic male sterile (TGMS) line Beijing Sterility 366 (BS366) has been utilized in hybrid breeding for a long time, but the molecular mechanism underlying male sterility remains unclear. Expression arrays, small RNA, and degradome sequencing were used in this study to explore the potential role of miRNA in the cold-induced male sterility of BS366. Microspore observation showed defective cell plates in dyads and tetrads and shrunken microspores at the vacuolated stage. Differential regulation of Golgi vesicle transport, phragmoplast formation, sporopollenin biosynthesis, pollen exine formation, and lipid metabolism were observed between cold and control conditions. Pollen development was significantly represented in the 352 antagonistic miRNA-target pairs in the integrated analysis of miRNA and mRNA profiles. The specific cleavage of *ARF17* and *TIR1* by miR160 and miR393 were found in the cold-treated BS366 degradome, respectively. Thus, the cold-mediated miRNAs impaired cell plate formation through repression of Golgi vesicle transport and phragmoplast formation. The repressed expression of *ARF17* and *TIR1* impaired pollen exine formation. The results of this study will contribute to our understanding of the roles of miRNAs in male sterility in wheat.

## 1. Introduction

Known as one of the most effective strategies to increase crop yield, heterosis has been widely used for crops [1]. Although male sterility is an unfavorable trait for an individual plant, it is a prerequisite for hybrid seed production and recurrent selection breeding [2]. Dozens of genic male sterile (GMS) genes have been cloned from maize, Arabidopsis, and rice, but only *Ms1*, *Ms2*, and *Ms5* have been cloned from wheat [3,4,5,6]. Thus, a deep understanding of the mechanism underlying male sterility in the TGMS line will be of great value for heterosis utilization.

MicroRNAs (miRNAs) are a class of ~21 nt non-coding RNAs that are important regulators ubiquitously existing in plants [7]. MiRNAs function in the posttranscriptional regulation of target gene expression via transcript cleavage or translational inhibition [8,9]. The involvement of miRNAs in plant reproductive organ development [10,11] and male sterility have been reported in plants [12,13]. Conserved miRNAs such as miR156, miR159, miR160, miR164, miR167, miR396, miR5200, and miR2275 were reported to be critical for reproductive development [14]. miR156 and its targets, the *squamosa promoter-binding protein-like* (*SPL*) genes, are highly conserved and regulate the vegetative-to-reproductive transition in plants [10]. Overexpression of soybean miR156b in Arabidopsis led to male sterility under heat stress [15]. miR159 targets *GAMYB*-*like* genes, resulting in anther defects or male sterility [16]. Overexpression of miR160 in cotton leads to anther indehiscence under high-temperature (HT) conditions, possibly due to the excessive activation of auxin signaling with the suppression of *ARF10* and *ARF17* [13]. In maize, zma-miR164 targets the orthologues of *OsDEX1* and *OsABCG15*, which are key functional genes in rice anther development [17,18]. In Arabidopsis, miR167 has been reported to be important for the fertility of both ovules and anthers by targeting *ARF6* and *ARF8* [19]. miR172 regulates *APETALA2* (*AP2*) and *AP2*-*like* genes. Overexpression of miR172 caused the conversion of sepals and petals into carpels and reduced the stamen number [20,21]. In addition, miR171 and miR396 are involved in floral development and male sterility by targeting *scarecrow*-*like* (*SCL*) and *growth regulatory factor* (*GRF*) genes [22,23].

To date, 77 mature miRNAs and 75 hairpin precursors have been registered in the miRBase for wheat (release 22). However, the microRNA population and its regulatory functions in wheat male sterility remain unclear [8,12,24]. Hence, it is of great application value and theoretical significance to explore the roles of miRNAs associated with male sterility. Beijing Sterility 366 (BS366) is a temperature-sensitive genic male sterile line (TGMS). It is male sterile at 12 °C (cold) and male fertile at 20 °C (control) with 12 h daylight. In this study, wheat genome expression arrays, small RNA, and degradome sequencing data from anthers of BS366 under control and cold conditions were used to explore the potential role of miRNA in wheat male sterility. Our results will provide novel insights into the roles of miRNAs in the male sterility of wheat.

## 2. Results

### 2.1. Pollen Development Was Defective in BS366 under Cold Conditions

**Figure 1:**
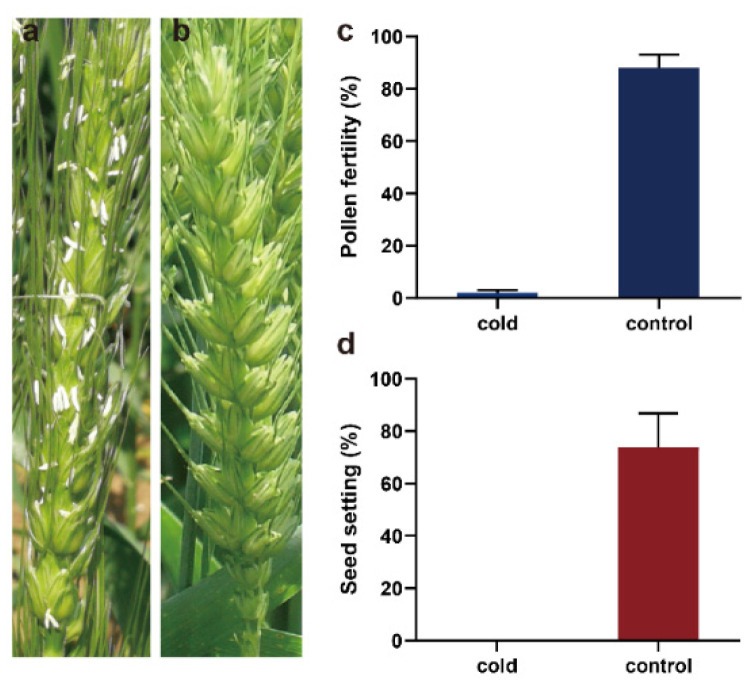
Phenotypic characterization of BS366 under cold and control conditions. Images of control- (**a**) and cold-treated (**b**) BS366 spikes at anthesis; the pollen fertility (**c**) and seed setting (**d**) of BS366 under cold and control conditions.

Wheat TGMS line BS366 was male fertile at 20 °C but sterile at 12 °C with 12 h of daylight. Both cold- and control-treated BS366 plants appear completely normal in growth habit and vigor before anthesis. Cold-treated BS366 has exposed anthers that are indehiscent compared with BS366 under control conditions (Figure 1a,b); nearly all the pollen in cold-treated BS366 was sterile compared with 88% in the control-treated BS366 (Figure 1a). Cold-treated plants are completely male-sterile and do not produce seed.

**Figure 2:**
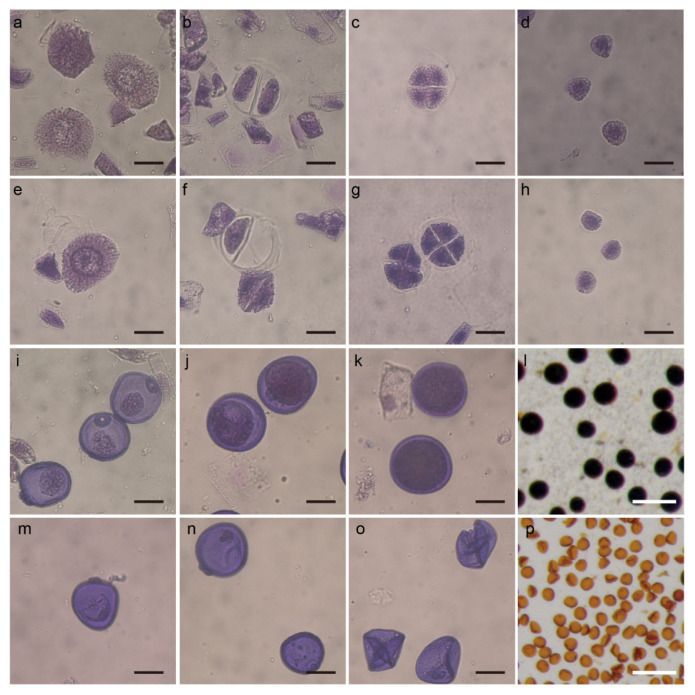
The microspores of BS366 under cold and control conditions. Microspores under control (**a**–**d**,**i**–**l**) and cold (**e**–**h**,**m**–**p**) conditions. Meiotic interphase (**a**,**e**), meiotic dyad (**b**,**f**), meiotic tetrad (**c**,**g**), early uninucleate stage (**d**,**h**), vacuolated stage (**i**,**m**), binucleate stage (**j**,**n**), and mature pollen stage (**k**,**o**). Pollen grains of BS366 under control (**l**) and cold (**p**) conditions stained with I_2_-KI. Bars in (**a**–**k**,**m**–**o**) 20 μm; bars in (**c**,**d**) 100 μm.

The morphology of BS366 pollen was examined at successive developmental stages under cold and control conditions. the pollen mother cells (PMCs) of BS366 under cold conditions was the same as that under control conditions (Figure 2a,e). However, severe defects were observed in some dyads and tetrads, with a defective cell plate in the cold-treated BS366 (Figure 2b,c,f,g). Dyads and tetrads that lacked smooth cell plates were unable to produce uninucleate pollen grains. Although pollen grains at the early uninucleate stages were normal for BS366 under cold conditions (Figure 2d,h), the pollen grains shrank severely from the vacuolated stage to the mature stage (Figure 2i,m). Compared with the control-treated BS366 (Figure 2j,k), no starch accumulated inside the microspore, and a smaller nucleus without generative and vegetative cells was observed (Figure 2n,o). The cold-treated pollen grains could not be stained by I_2_-KI like the normal pollen (Figure 2l,p).

### 2.2. Construction of the Co-Expression Network for All of the Expressed Genes

**Figure 3:**
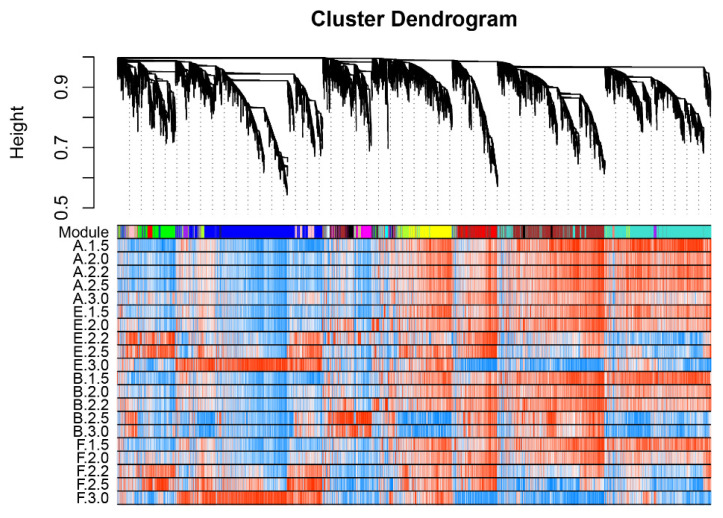
Clustering dendrograms and expression heatmap of all the expressed probes obtained via hierarchical clustering of topological overlapping dissimilarity. A total of 18 co-expression modules were constructed and are shown in different colors. Blue and red in the heatmap indicate low and high expression, respectively.

To understand the gene regulatory network and identify genes responding to cold treatment in BS366, weighted gene co-expression network analysis (WGCNA) algorithms were carried out for all of the expressed probes. In total, 16,246 expressed probes were used to construct co-expression modules. The flashClust tools package was used to perform the cluster analysis (Figure S1). Before WGCNA, network topology analysis for various soft thresholding powers was carried out to determine the candidate power values for relative, balanced scale independence, and mean connectivity in WGCNA. As shown in Figure S2, the scale independence reached 0.85 when the power value was 13. Thus, a power value of 13 was selected to produce a hierarchical clustering tree for the expressed probes (Figure 3). Finally, 16,318 probes were assigned to 18 expression modules. The number of probes for each module ranged from 50 to 3813 (Table S1). The correlation of different modules is shown in Figure S3. Among the 18 modules, probes in blue, brown, yellow, and turquoise made up the majority (72%) of all the expressed probes.

### 2.3. Expression and Function Analysis of Genes in the Blue and Brown Modules

**Figure 4:**
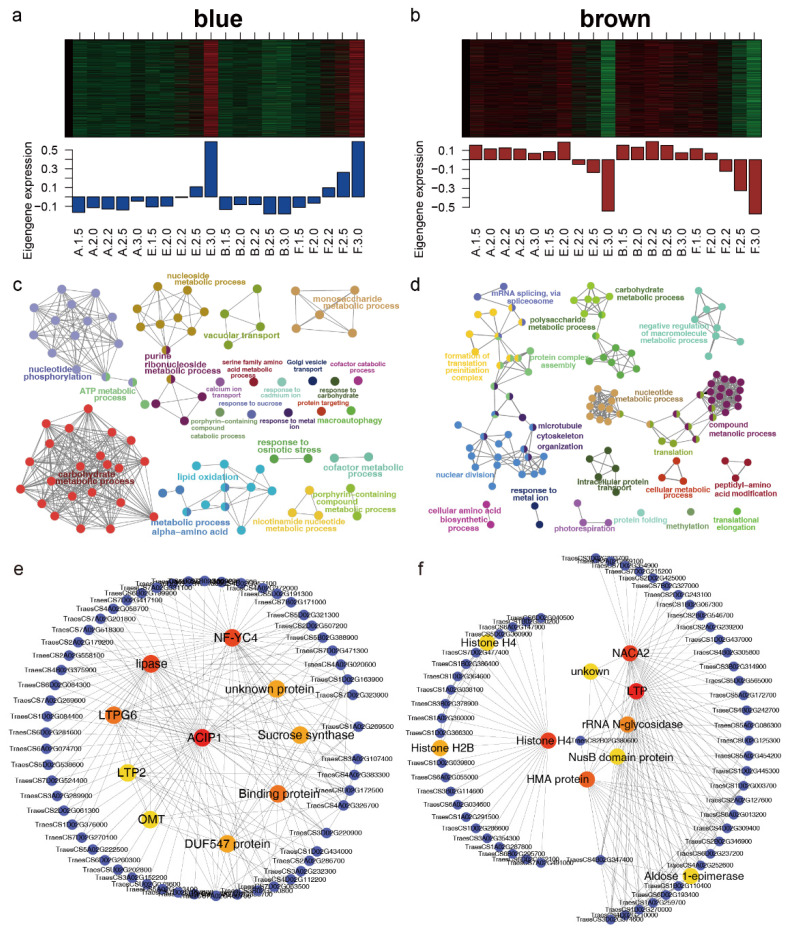
Expression and gene ontology (GO) analysis of genes in the blue and brown modules. Gene expression pattern in the blue (**a**) and brown (**b**) modules: In the heatmap, red indicates upregulated genes, black indicates neutral genes, and green indicates downregulated genes. The bar plots show the eigengene values. The GO analysis of genes in the blue (**c**) and brown (**d**) modules: All of the biological processes were fused using ClueGO, and processes with similar functions were clustered into 25 (**c**) and 20 (**d**) clusters. The names of the significantly represented clusters are shown in larger and colored fonts. Cycles with different colors indicate that the GO term can be associated with different groups. The other GO terms are shown in smaller and gray font. Gene co-expression network of genes in the blue (**e**) and brown (**f**) modules: The top 200 connections are shown for each module. Genes with the top 10 connections are shown in larger sizes and are colored red to yellow. Red and yellow indicate higher and lower numbers of interactions between the genes, respectively, and orange indicates the median number of interactions of genes.

The expression of genes assigned to the blue and brown modules was opposite. There were 3506 probes assigned to the blue module, in which probes were suppressed under cold (A/B) conditions but induced under control (E/F) conditions during stages 2.2, 2.5, and 3.0 (Figure 4a). In total, 3105 probes were assigned to the brown module, in which probes were expressed at higher levels during stages 2.2, 2.5, and 3.0 under cold (A/B) conditions than under control (E/F) conditions (Figure 4b). After converting the probe IDs to gene IDs, gene ontology (GO) analysis was carried out for the genes assigned to both modules. Genes in the blue module were mainly enriched in biological processes such as carbohydrate metabolic process, ATP metabolic process, nucleotide phosphorylation, vacuolar transport, Golgi organization, and lipid oxidation (Figure 4c and Supplementary Table S2). As shown in Figure 4d and Supplementary Table S2, biological processes including mRNA splicing via spliceosome, microtubule cytoskeleton organization, nuclear division, and nucleobase-containing compound metabolic process, were significantly represented for genes in the brown module. The correlations of the genes in both modules were used to construct the co-expression network. Networks for the top 200 correlations were constructed using ClueGO in Cytoscape. In the blue module, three genes involved in lipid metabolism (*LTPG6*, *lipase*, and *LTP2*), one gene (*ACIP1*) encoding a microtubule-associated protein, and one gene encoding a NF-YC4 transcription factor were among the top ten hub genes (Figure 4e). In the brown module, genes encoding three histone proteins, one LTP protein, and one NAC transcription factor were among the top ten hub genes (Figure 4f).

### 2.4. The Differentially Expressed Genes between Cold and Control Conditions

**Figure 5:**
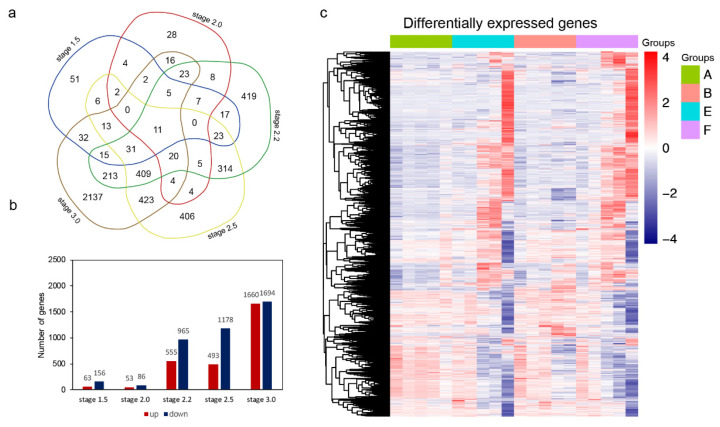
Differentially expressed genes between cold and control conditions. (**a**) Venn diagram of all the differentially expressed genes at five stages. (**b**) Number of differentially expressed genes that were up- or downregulated at each stage. Up means upegulated under cold conditions compared to the control conditions at respective stages. (**c**) Hierarchical cluster analysis of all differentially expressed genes.

To explore the expression changes of genes under cold conditions, the differentially expressed genes (DEGs) between A/E and B/F were identified for five development stages. The common DEGs for A vs. E and B vs. F comparisons were identified. There are 231, 164, 1581, 1897, and 3412 DEGs common to the A vs. E and B vs. F groups at five successive stages (Figure 5a). The number of downregulated genes was greater than that of upregulated genes at all five stages except stage 3.0. The number of DEGs between cold and control conditions increased noticeably from the 2.2 stage (Figure 5b). A heatmap of all the DEGs between cold and control conditions at five stages is shown in Figure 5c. These results may indicate that three later stages may be more important for the pollen viability of BS366. Thus, GO and KEGG analysis were carried out for DEGs at these development stages.

**Figure 6:**
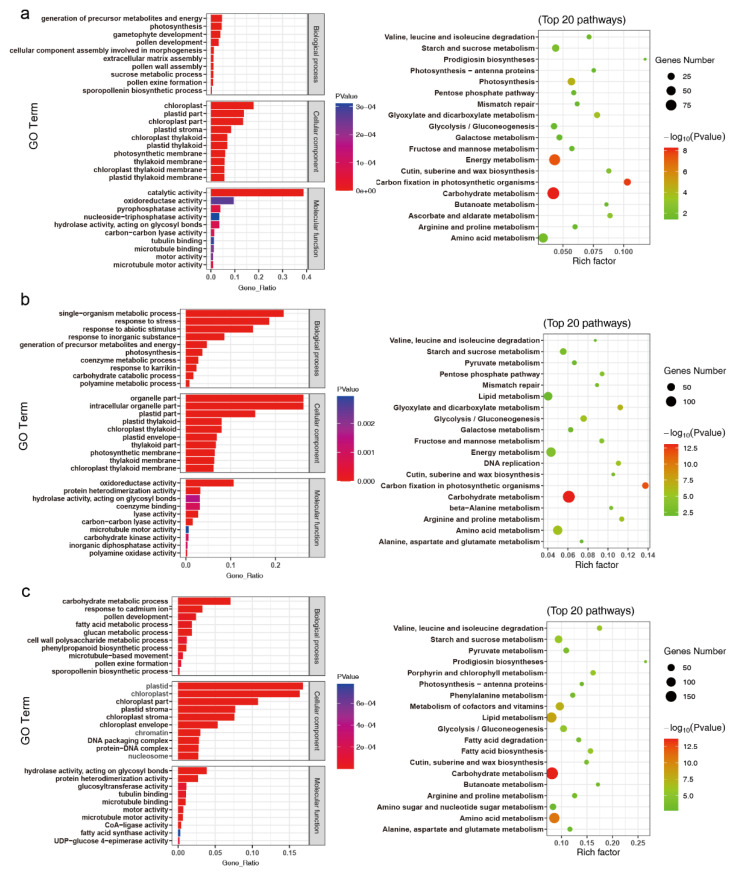
Functional analysis of differentially expressed genes between cold and control conditions. GO and KEGG analysis of DEGs between cold and control conditions at stages 2.2 (**a**), 2.5 (**b**), and 3.0 (**c**). Left, GO enrichment analysis; right, KEGG analysis.

As shown in Figure 6a, the pollen and anther cutin development processes differed between cold and control conditions as early as stage 2.2. Biological processes such as the sporopollenin biosynthetic process, pollen exine formation, and pollen wall assembly were significantly represented at stage 2.2. KEGG analysis revealed that the enrichment of cutin, suberine, and wax biosynthesis pathway was first observed at stage 2.2. Aside from processes mentioned above, phragmoplast and cell plate formation-related processes, such as microtubule-based movement, microtubule motor activity, motor activity, microtubule binding, and tubulin binding were also first observed at stage 2.2. The significant enrichment of the lipid metabolism pathway was first observed at stage 2.5 in the KEGG analysis. Biological processes such as the fatty acid metabolic process, fatty acid synthase activity, and CoA-ligase activity were significantly represented at stage 3.0. KEGG analysis revealed that pathways involved in lipid metabolism, fatty acid biosynthesis, and fatty acid degradation were significantly represented at stage 3.0.

### 2.5. Differential Expression of Genic Male Sterility Genes

**Figure 7:**
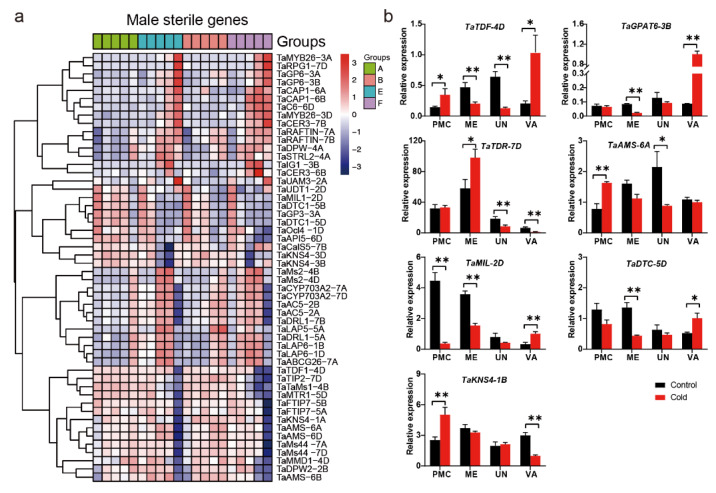
The expression and validation of genic male sterility genes. (**a**) Heatmap of the differentially expressed male sterility genes during three developmental stages. (**b**) qRT–PCR analysis of the selected male sterility genes. Asterisks indicate significant differences between cold and control conditions (Student’s *t*-test, * *p*-value < 0.05, ** *p*-value < 0.01). PMC, pollen mother cell stage; ME, meiosis stage; UN, uninucleate stage; VA, vacuolated stage.

To date, more than one hundred GMS genes have been cloned from other plants [25]. The expression patterns of wheat orthologues of these GMS genes were analyzed in this study. For example, *TaTDF1*-*4D* encodes an R2R3-MYB transcription factor; *TaMMD1-4D* encodes a PHD domain-containing protein; and *TaUDT-1D*, *TaAMS-6A*, *TaAMS-6B*, and *TaAMS-6D* encode bHLH transcription factors. *TaUDT1-2D* was significantly repressed by cold treatment during stage 3.0, while *TaTDF1-4D* and *TaMMD1-4D* were induced by cold treatment during stage 3.0. The expression of *TaAMS-6A* and *TaAMS-6B* was downregulated at stage 1.5 but upregulated at stage 3.0 under cold conditions. *TaMMD1-3D* was only induced by cold treatment during stage 3.0. The expression of *TaGPAT6-3B* was repressed during the two late stages, but *TaGPAT6-3A* was only suppressed during stage 3.0. *CalS5* encodes a callose synthase [26], and *KNS4* encodes a type II arabinogalactan β-(1,3)-galactosyltransferase [27]. *RPG1* encodes a sugar transporter in Arabidopsis [28]. In this study, *TaCalS5-7B*, *TaKNS4-3B*, and *TaKNS4-3D* were all induced during stages 2.5 and 3.0, but *TaRPG1-7D* was repressed during stages 2.5 and 3.0 under cold conditions (Figure 7a). The expression of several GMS genes was validated using quantitative real-time PCR (qRT–PCR) during the pollen mother cell (PMC) stage, meiosis stage, uninucleate stage, and vacuolated stage under both cold and control conditions. The expression of *TaTDF*-*4D* was significantly induced at both the PMC and vacuolated stage but repressed at the meiosis and uninucleate stages. *TaTDR*-*7D* was significantly induced at the meiosis stage but repressed at two latter stages. The expression of *TaGPAT6*-*3B* and *TaDTC*-*5D* was repressed at the meiosis stage but induced at the vacuolated stage. *TaMIL*-*2D* was significantly repressed at two early stages, but finally induced at the vacuolated stage under cold conditions. Both *TaKNS4*-*1B* and *TaAMS*-*6A* were significantly induced at the PMC stage, but they were repressed under cold conditions at the vacuolated and uninucleate stages, respectively. (Figure 7b).

### 2.6. Mapping of sRNA Reads to the Reference Genome

After filtering and quality control of the raw reads, a total of 77,531,794 clean tags were generated for seven libraries, with an average of 11,075,970 reads per library. Reads from seven libraries were mapped to the wheat genome (IWGSC RefSeq v1.1), and the underlying gene annotation was used to assign reads to the respective gene models (ftp://ftp.ensemblgenomes.org/pub/plants/release-44/gff3/triticum_aestivum, accessed on 12 August 2020). Finally, 87.51% (77,531,794/67,862,447) of the reads were mapped to the genome. A total of 31,670,965 reads were uniquely mapped to the genome, with a unique mapping rate of 33.98% (Table S3). The length distribution of the tags across all of the libraries was analyzed. The most abundant siRNAs were 21- and 24-nt lengths, with the highest percentages of 25.39% and 65.79% in libraries T0 and A1.5, respectively (Figure S4). All of the aligned read sequences were then annotated by reference to the Rfam database (version 11.0, http://rfam.xfam.org/, accessed on 12 August 2020) and GenBank (https://www.ncbi.nlm.nih.gov/, accessed on 12 August 2020), which allowed for the removal of rRNA, tRNA, snRNA, and snoRNA sequences (Figure S5); the remaining tags were used to query the wheat and other plant miRNAs in miRBase.

Finally, this analysis yielded 510 already documented miRNAs (known miRNAs), including 108 wheat miRNAs and 402 miRNAs in other plants. There were 413 and 420 known miRNAs expressed in cold (A) and control conditions (E), respectively (Figure S6). Under cold conditions, 270 known miRNAs were commonly expressed during stages 1.5, 2.2, and 3.0. There were 19, 36, and 37 known miRNAs specific to stages 1.5, 2.2, and 3.0, respectively. Under control conditions, 250 known miRNAs were expressed during all three stages. There were 30, 49, and 28 known miRNAs exclusively expressed during stages 1.5, 2.2 and 3.0, respectively. Among the top 100 expressed miRNAs, there were miRNAs from the miR156, miR159, miR165, miR166, miR167, miR171, miR172, miR2118, miR319, miR396, tae-miR156, tae-miR159, tae-miR164, tae-miR167, and tae-miR171 miRNA families (Supplementary Table S4). These results indicate that these highly expressed miRNAs may be involved in anther development in wheat. After blasting against miRNAs in miRBase, reads with no homology to any previously known miRNA/pre-miRNA were used to predicate the novel miRNAs using MIREAP. Finally, 4478 novel miRNAs were predicted in seven libraries, including 4264, 4206 and 3797 for the cold, control, and T0 samples, respectively (Figure S6). Under cold conditions, there were 2554 novel miRNAs expressed in common during the three stages, with 113, 211, and 252 specific to stages 1.5, 2.2, and 3.0, respectively. In the control samples, there were 2312 novel miRNAs expressed in common during the three stages, with 246, 250, and 231 specific to stages 1.5, 2.2, and 3.0, respectively (Figure S6).

### 2.7. Differentially Expressed miRNA Identification, Target Prediction and Function Analysis

**Figure 8:**
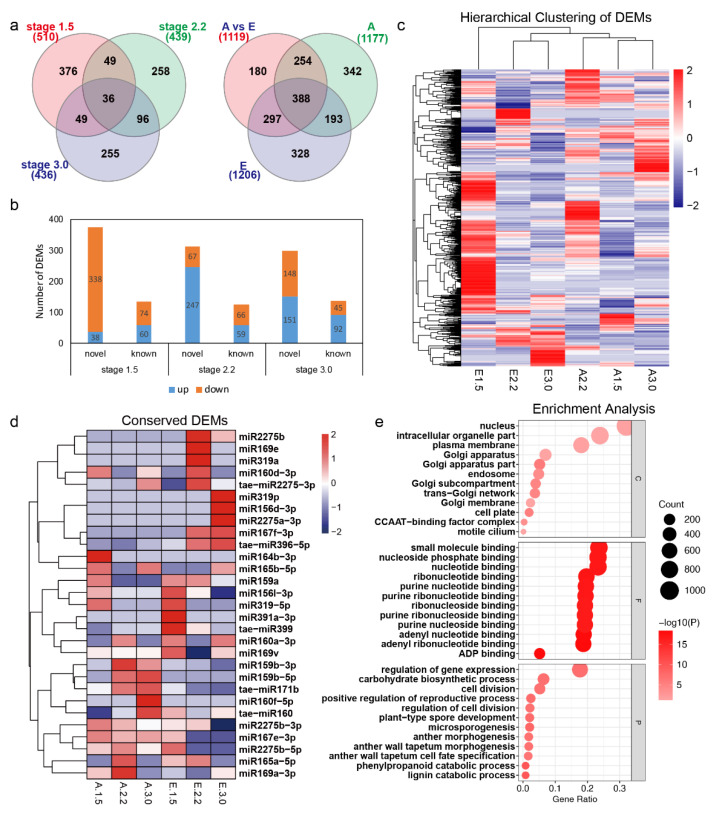
Differentially expressed miRNAs between cold and control conditions at the respective stages. (**a**) Venn diagram of the differentially expressed miRNAs at stages 1.5, 2.2, and 3.0 (left); Venn diagram of the differentially expressed miRNAs in cold and control conditions, and the DEMs between the cold and control conditions. (**b**) The up- and downregulation of DEMs between cold and control conditions. (**c**) Expression heatmap for all DEMs in/between cold and control conditions. (**d**) Heatmap of the conserved known DEMs mentioned. (**e**) GO analysis of the predicted targets for the DEMs between cold and control conditions. A and E in (**a**) represent cold and control; C, F, and P in (**e**) indicate cellular component, molecular function, and biological process, respectively.

To identify cold-responsive miRNAs during anther development, the expression levels of the miRNAs were compared within/between the cold and control conditions. The differentially expressed miRNAs (DEMs) were processed using the criteria of |fold change| > 2 and *p* value < 0.05. In total, 1177 and 1206 DEMs were identified between adjacent stages for cold and control conditions, respectively. There were 1119 DEMs between the cold and control conditions for the three stages. There were 388 DEMs common to the three sections, with 328, 342, and 180 DEMs specific to the control, cold and cold vs. control sections, respectively (Figure 8a). A large proportion of DEMs (939/1119) between cold and control conditions were also differentially expressed under cold or control conditions. There were 510, 439, and 436 DEMs between the two conditions during stages 1.5, 2.2, and 3.0, respectively (Figure 8a). The number of DEMs upregulated under cold conditions during the three stages varied, with stage 2.2 having the most DEMs (306) and stage 1.5 having the fewest DEMs (98) (Figure 8b). Compared with 417 DEMs during stage 1.5, only 133 DEMs and 193 DEMs were downregulated during stages 2.2 and 3.0. The expression heatmap showed that three samples under cold conditions clustered together and differed from the 2.2 and 3.0 stages under control conditions (Figure 8c). Several conserved miRNAs were found to be differentially expressed between the two conditions. For example, miR160a-3p and miR160d-3p were consistently differentially expressed during all three stages. The expression of miR2275b significantly differed from that of miR2275b-5p during stages 2.2 and 3.0. Compared to the control conditions, miR319-5p and miR167e-3p were induced, while miR159a and miR167f-3p were repressed during stages 2.2 and 3.0 under cold conditions (Figure 8d). miR164b-3p showed higher expression levels during stage 1.5 under cold conditions. The expression levels of miR319a and miR319p were higher during stages 2.2 and 3.0 under cold conditions, respectively. 

The candidate target prediction was processed using psRobot (http://omicslab.genetics.ac.cn/psRobot/, accessed on 12 August 2020) and TargetFinder (http://jcclab.science.oregonstate.edu/node/view/56334, accessed on 12 August 2020). Targets predicted by both methods were used as the final targets for miRNAs. Among the 1119 DEMs between two conditions, 887 miRNAs were found to have corresponding target genes. In total, 13,026 miRNA-target pairs were found, corresponding to 7158 genes. Among the DEMs with target genes, 738 of the 887 genes had more than one target. A total of 3068 of 7160 targets were predicted to be targeted by more than one miRNA. A total of 4604 miRNA-target pairs were identified for the known miRNAs, corresponding to 3555 candidate genes and 201 known DEMs (Supplementary Table S5).

To gain an overview of the function of the DEMs between cold and control conditions, GO analysis was carried out for the 3555 targets. Biological processes including lignin catabolic process, phenylpropanoid catabolic process, microsporogenesis, and anther wall tapetum morphogenesis were significantly represented. In the cellular component category, Golgi apparatus-related GO terms were represented. Cellular components such as the cell plate, CCAAT-binding factor complex, and nucleus were significantly represented (Figure 8e). All of these results indicate that miRNAs may mediate male sterility in BS366 through regulating the cell plate, Golgi network, lignin catabolic process, phenylpropanoid catabolic process, and anther wall tapetum morphogenesis.

To study the potential interconnection between conserved known DEMs, expression correlation analysis (Pearson correlation) was carried out for those miRNAs mentioned above. As shown in Figure S7, high correlation was observed between some miRNA families. The miR2275, miR319, and miR156 families are highly correlated with all the miRNA families in this analysis (|Pearson correlation coefficient| > 0.8). The miRNAs from the miR159 family were highly correlated with miRNAs from the miR156, miR165, miR169, miR2275, and miR319 families. The miRNAs from the miR160 family were highly correlated with miRNAs from the miR169, miR2275, and miR319 families. The miRNAs of the miR165 family are highly correlated with all the miRNA families except miR167, miR391, miR171, and miR396. miR167 was highly correlated with the miR2275 and miR319 families. Expression of miRNAs including miR159a, miR319-5p, miR167e-3p, and miR2275b-5p decreased at the late stages. These results suggested that the expression of those miRNAs might be repressed by other miRNAs. As shown in Figure S7, miR159a was negatively correlated with miR156d-3p, miR165b-5p, miR2275a-3p, and miR319p. The expression of miR2275b-5p was negatively correlated with that of miR167f-3p, miR2275b, tae-miR2275-3p, and tae-miR396-5p. miR167e-3p was negatively correlated with miR2275b, tae-miR2275-3p, and tae-miR396-5p. Thus, we may conclude that a potential interconnection among those miRNAs exists.

### 2.8. The Antagonistic miRNA–Target Pairs Identified during Three Stages

**Figure 9:**
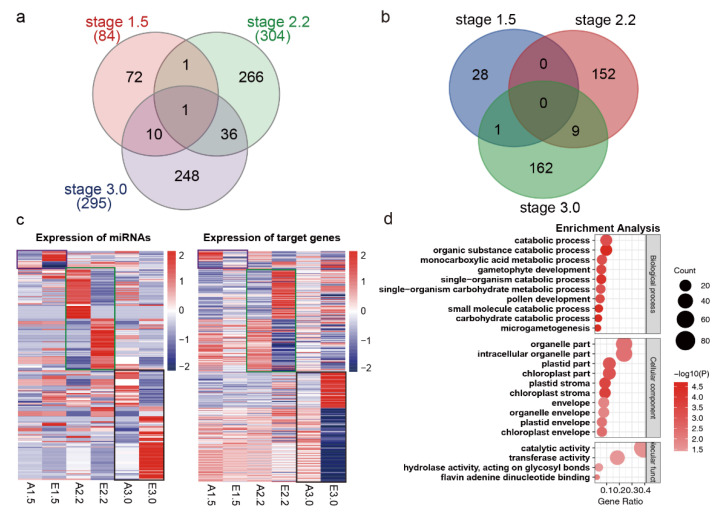
The predicted opposite miRNA–mRNA pairs and functional analysis. (**a**) Differentially expressed miRNA–target pairs identified in the array and miRNA sequencing data. (**b**) Heatmap of the differentially expressed miRNA–target pairs with opposite expression patterns. miRNAs and genes with opposite expression patterns were framed using the same color in the heatmap for miRNAs and genes. The purple, red, and black frames represent opposite miRNAs and target genes at stages 1.5, 2.2, and 3.0, respectively. (**c**) Venn diagram of the predicted opposite miRNA–mRNA pairs at three developmental stages. (**d**) GO analysis of the target genes from the opposite miRNA–mRNA pairs.

To explore the role of miRNAs in the expression of their candidate target genes, an integrated analysis was performed for the miRNA sequencing and array expression data. The differentially expressed targets and DEMs were identified during respective stages. Finally, 634 differentially expressed miRNA–target pairs (both the targets and miRNAs were differentially expressed) were found, with 84, 304, and 295 miRNA–target pairs for stages 1.5, 2.2, and 3.0, respectively (Figure 9a and Supplementary Table S6). miRNAs usually work by cleaving their targets; thus, the expression of miRNAs and their corresponding target genes would be negatively correlated. Finally, 352 antagonistic miRNA–target pairs were identified, with 29, 161, and 172 pairs for stages 1.5, 2.2, and 3.0, respectively (Figure 9b and Supplementary Table S6). More antagonistic miRNA–mRNA pairs were observed for stages 2.2 and 3.0. Only one miRNA–target pair was in common during stages 1.5 and 3.0, but nine common miRNA–target pairs were found for stages 2.2 and 3.0. The expression patterns of all of the antagonistic expressed miRNA–target pairs are shown in Figure 9c. Finally, 138 DEMs and 206 putative target genes were included to form 352 negative miRNA–target pairs (Supplementary Table S6). GO analysis of the 206 targets revealed that biological processes such as carbohydrate catabolic process, microgametogenesis, gametophyte development, and pollen development were significantly represented (Figure 9d and Supplementary Table S7).

**Figure 10:**
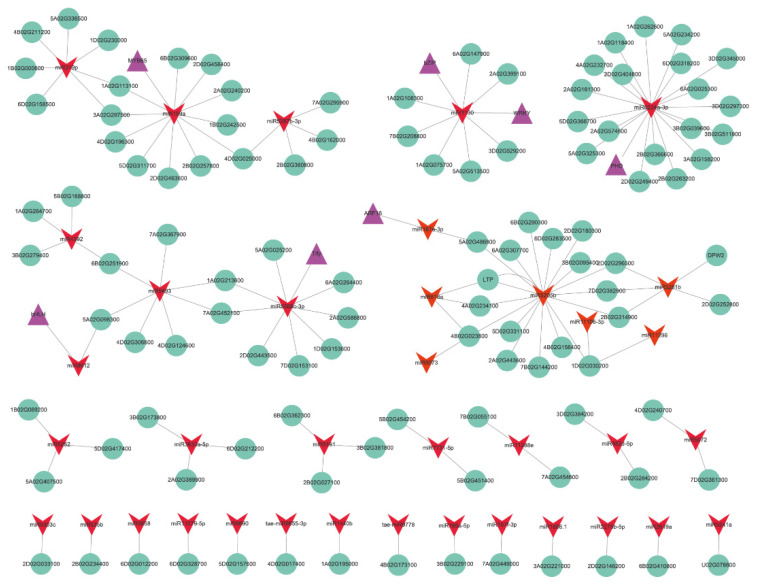
The predicted opposite miRNA–mRNA pairs for known DEMs between the cold and control conditions. Red V-shape represents miRNAs; green circle represents genes; purple triangle represents transcription factors.

In the network of genetic regulation in organisms, the role of TFs is to regulate mRNA transcription, while the function of miRNAs is to regulate the stability of mRNAs and affect their translation after transcription. Among the 352 opposite miRNA–target pairs, miRNA–TF pairs were identified for three stages (Figure 10 and Supplementary Table S6). At stage 3.0, upregulated miR167e-3p repressed the expression of *ARF16* (TraesCS7D02G161900); the expression of the *Tify transcription factor* (TraesCS4D02G295900) was repressed by the upregulated miR5568c-3p; downregulation of miR1130 induced the expression of genes encoding WRKY34 (TraesCS4A02G193600) and bZIP (TraesCS2B02G113200). The downregulation of miR6224a-3p and miR8612 induced the expression of *PHD transcription factor* (TraesCS2A02G312700) and *bHLH transcription factor* (TraesCS2B02G289900), respectively. Downregulated novel-m1862-3p induced the expression of a *TaGAMYB*-*like* gene, an orthologue of *HvGAMYB* (TraesCS3A02G336500) at stages 1.5 and 3.0. In addition, the downregulated miR159a induced the expression of *TaMYB65* (TraesCS1D02G307500, an orthologue of *MYB65* in Arabidopsis) during stage 2.2.

**Figure 11:**
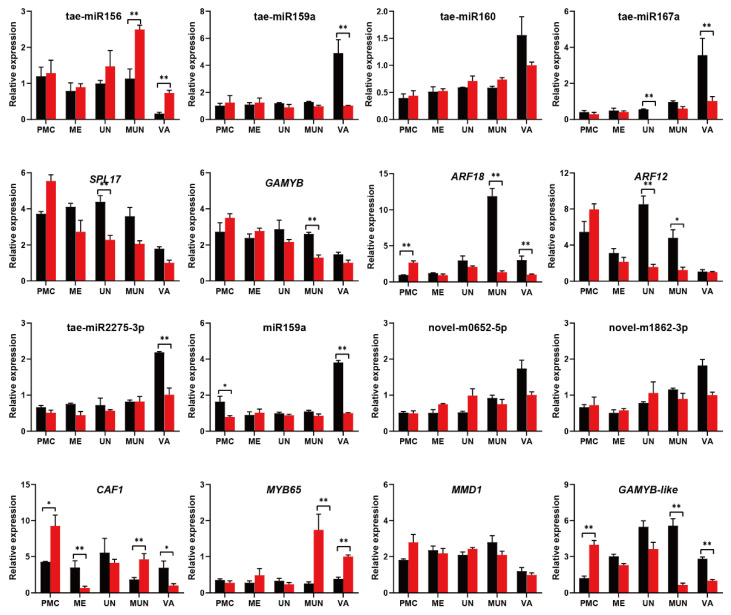
Expression of conserved miRNAs and target genes at different developmental stages between cold and control conditions. F indicates control conditions, S indicates cold conditions. Asterisks indicate significant differences between cold and control conditions (Student’s *t*-test, * *p*-value < 0.05, ** *p*-value < 0.01). PMC, pollen mother cell stage; ME, meiosis stage; UN, uninucleate stage; MUN, middle uninucleate stage; VA, vacuolated stage.

Orthologues of GMS genes with potential roles in male sterility were also found in the negative miRNA–target pairs (Supplementary Table S6 and Figure 10). *TaDPW2* (TraesCS2B02G215000, an orthologue of *OsDPW2*) was negatively regulated by miR5281b during stage 1.5. During stage 3.0, *TaMMD1* (TraesCS4D02G050000, an orthologue of *MMD1* in Arabidopsis) was negatively regulated by novel-m0652-5p. The expression of several miRNAs together with their target genes was studied using qRT–PCR (Figure 11). The expression of tae-miR156 was induced at all stages, but significantly induced at the middle uninucleate stage. The expression of *SPL* was repressed from the meiosis stage, but significantly repressed at the uninucleate stage. tae-miR160 and tae-miR167e were repressed at the vacuolated stage, but their putative target genes *GAMYB* and *ARF12* were both repressed at earlier stages. The expression of tae-miR160 was a little higher under cold conditions than under control conditions from the uninucleate stage, but *ARF18* was significantly repressed from the middle uninucleate stage. tae-miR2275-3p was repressed only during the vacuolated stage; its putative target genes were induced during the PMC and middle uninucleate stages but repressed under meiosis and vacuolated stages. The expression of miR159 was significantly repressed under cold conditions during the PMC and vacuolated stages, but its putative target gene *MYB65* was significantly induced during the middle and vacuolated stages. The expression of novel-miR0652 and novel-miR1862 differed in the expression data, but not in qRT–PCR validation. The putative target of novel-miR1862, *GAMYB*-*like* was induced at the PMC stage, but repressed at the middle and vacuolated stages.

### 2.9. Identification of miRNA Target Genes via Degradome Sequencing

Target gene validation is important to further understand the biological functions of miRNAs. To identify the targets cleaved by the candidate miRNAs identified in the present study, degradome sequencing was used for the cold- and control-treated BS366 anthers. In total, 36,647,546 and 27,244,395 raw reads were obtained from DF (degradome for anthers under fertile condition) and DS (degradome for anthers under sterile condition), respectively. After removing the reads < 15 nt and adaptors, 35,971,562 and 26,622,338 unique reads from the DF and DS libraries, respectively, were mapped to the wheat genome. The cleaved targets for miRNAs were identified based on a method in the CleaveLand pipeline [29]. The sliced target transcripts were categorized into five categories (0, 1, 2, 3, and 4) according to the relative abundance of the tags at the target sites. Targets in category 0 were evaluated as the most significant.

**Figure 12:**
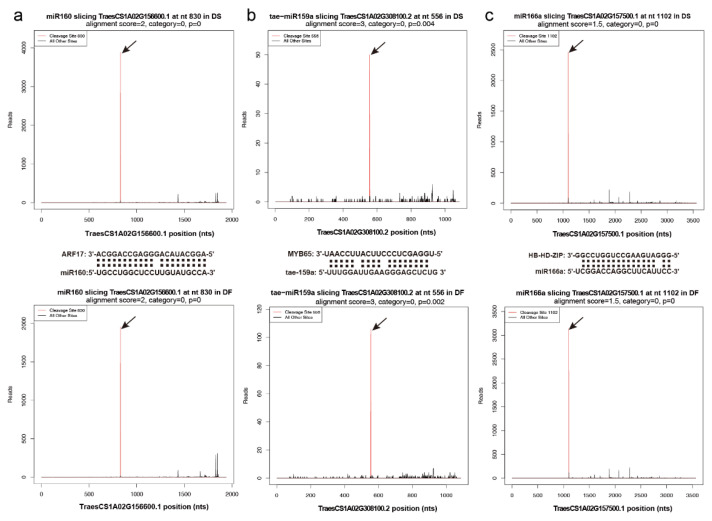
T-plots of representative miRNA targets under cold and control conditions. (**a**) The T-plots of *ARF17* targeted by miR160. (**b**) The T-plots of *MYB65* targeted by tae-159a. (**c**) The T-plots of *HB*-*HD*-*ZIP* targeted by miR166a. Upper panel, cold conditions; lower panel, control conditions. The red line represents the sliced target transcripts and is indicated by an arrow. The alignments show the miRNA with a portion of its target sequence (middle panel).

In our degradome dataset, 495 target transcripts for 572 known miRNA families were identified in the two libraries, corresponding to 1141 miRNA-target cleavage pairs. Of the 495 target transcripts, 115 (23.23%) were found in both libraries, 355 (71.72%) were found only in the DS library, and 41 (8.28%) were found only in the DF library (Supplementary Table S8). As expected, most of the transcripts targeted by the highly conserved miRNAs were associated with conserved target genes. miR160 targeted the *ARF17* gene (TraesCS1A02G156600) in both DS and DF libraries. The transcript levels of ARF17 were higher in the DS than those in the DF library (Figure 12a). Tae-miR159a targeted *MYB65* (TraesCS1A02G308100) in both DS and DF libraries. The reads of *MYB65* were more abundant in the DF library than in the DS library (Figure 12b). miR166 targeted *HB*-*HD*-*ZIP* genes (TraesCS1B02G173900, TraesCS1A02G157500) in both DS and DF libraries. The reads of *HD*-*Zip* were more abundant in the DF library than in the DS library (Figure 12c).

**Figure 13:**
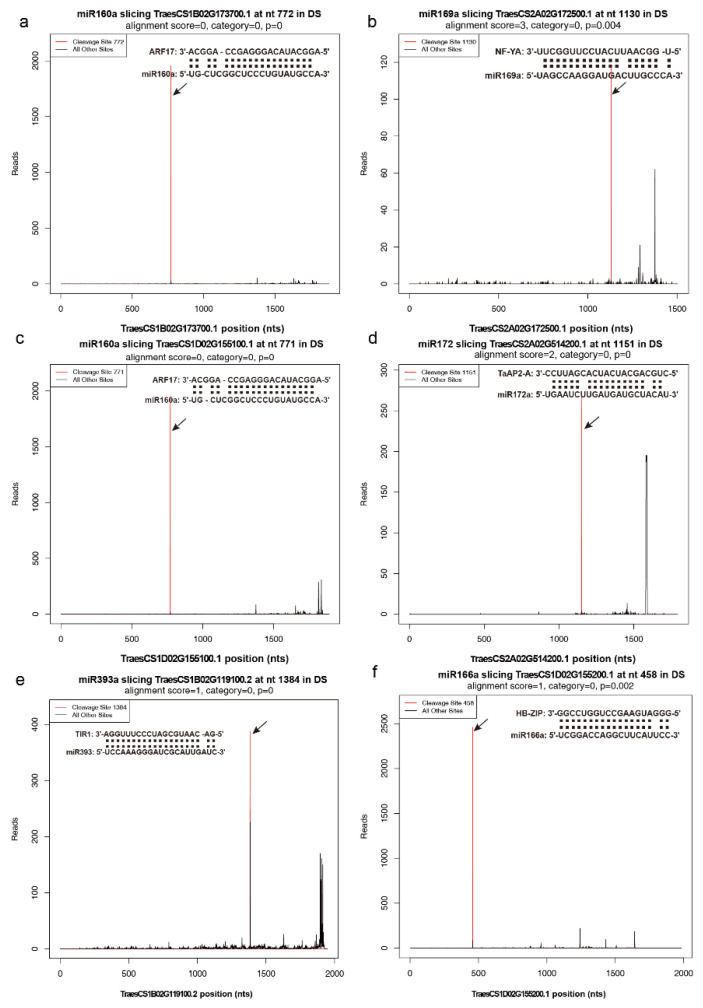
T-plots of representative miRNA targets under cold conditions. (**a**) The T-plots of *ARF17* targeted by miR160a. (**b**) The T-plots of *NF-YA* targeted by miR169a. (**c**) The T-plots of *ARF17* targeted by miR160a. (**d**) The T-plots of *TaAP2*-*A* targeted by miR172. (**e**) The T-plots of *TIR1* targeted by miR393. (**f**) The T-plots of *HD-ZIP* targeted by miR66a. The red line represents the sliced target transcripts and is indicated by an arrow. The alignments show the miRNA with a portion of its target sequence.

There are genes that could only be cleaved by corresponding miRNAs in the DS library. The cleavage of *ARF17* genes (TraesCS1B02G173700, TraesCS1D02G155100) was only observed in the DS library (Figure 13a,c). An NF-YA transcription factor encoding gene (TraesCS2A02G172500) was targeted by miR169a only in the DS library (Figure 13b). *TaAP2-A* (TraesCS2A02G514200) was targeted by miR172 only in the DS library (Figure 13d). Two TIR1 genes (TraesCS1B02G119100 and TraesCS1D02G099900) were cleaved by miR393 only under cold conditions (Figure 13e). A HD-Zip coding gene (TraesCS1D02G155200) was cleaved by miR166a under cold conditions (Figure 13f).

## 3. Discussion

### 3.1. The Potential Role of Conserved miRNAs in the Anther Development of Wheat

The fertility of TGMS lines is impaired by temperature. It has been reported that epigenetic regulation, such as DNA methylation and miRNA regulation, participates in male sterility in plant TGMS lines [8,30]. Several conserved miRNAs have been reported to be essential for reproductive development in plants, including miR156, miR159, miR160, miR164, miR165, miR167, miR169, miR319, miR2275, tae-miR160, tae-miR171, tae-miR396, and tae-miR2275 [14] (Figure 7). In this study, miR159 was found to be highly abundant and differentially expressed between cold and control conditions during stage 2.2. The miR159-GAMYB pathway is conserved in higher plants, where *GAMYB* expression promotes programmed cell death in seeds (aleurone) and anthers (tapetum) [31]. The closely related *MYB33* and *MYB65* genes of Arabidopsis have high sequence similarity to the barley (*Hordeum vulgare*) *GAMYB* gene. T-DNA insertional mutants were isolated for both genes, and an *myb33myb65* double mutant was defective in anther development [32]. In Arabidopsis, miR159 is a three-member gene family whose mature products differ by a single nucleotide. The three targets of miR159-*MYB33*, *MYB65*, and *MYB101* have been reported to be involved in vegetative to reproductive transition, anther development, male-specific cytokinesis, and programmed cell death (PCD) [32,33]. In this study, miR159a was predicted to negatively regulate *GAMYB transcription factor* (TraesCS1D02G307500), an orthologue of *MYB domain protein 65* (*MYB65*) in Arabidopsis. The cleavage of *TaMYB65* was validated in the degradome sequencing. These results suggest that miRNA159a-MYB65 may also have a function in BS366 male sterility. miR172 regulates *APETALA2* (*AP2*) and *AP2*-*like* genes. The role of miR172 in the regulation of flowering time and floral organs has been reported in Arabidopsis [20]. The cleavage of *TaAP2*-*1A* was only found in the cold-treated degradome. This result suggested that miR172-*AP2* may have a function in the male sterility of BS366.

### 3.2. Impaired Cell Plate Formation during Meiosis

Cellular trafficking is the foundation of cellular morphology and function. The Golgi apparatus plays an important role in the secretion and transportation of cellular vesicles during cellular trafficking [34]. During cytokinesis in plants, the physical insertion of a membranous cell plate depends on the cytokinetic apparatus of the phragmoplast [35]. It has been reported that Golgi-derived vesicles transport xyloglucans and other cell wall-containing materials to form the phragmoplast [36]. In this study, a defect in sterile BS366 was first observed during the dyad stage, with abnormal cell plate formation (Figure 2). A disrupted dynamic organization of phragmoplast microtubules and deposition of the cell plate under sterile conditions was observed in sterile BS366 at meiosis [37]. In the blue module, vesicle-mediated transport and Golgi vesicle transport were significantly repressed. The DEMs between cold and control conditions were mainly involved in Golgi vesicle transport, Golgi-associated vesicle membrane, and phragmoplast. The expression of putative vesicle-associated protein 2 was predicted to be repressed by miR6224a-3p during the 3.0 stage. Thus, miRNAs may play a critical role in the defective dyad and tetrad. Phragmoplasts in plants are composed of microtubules (MTs), microfilaments, motor proteins, and several regulators [38]. In the brown module, genes involved in microtubule cytoskeleton organization were induced under cold conditions (Figure 4). One gene (ACIP1) encoding a microtubule-associated protein was one of the hub genes in this module (Figure 4). Genes involved in tubulin binding, microtubule binding, and motor activity were differentially expressed between cold and control conditions from stage 2.2 (Figure 6). A *Tubulin 1* was targeted by miR5340 and miR5293 in the DS library. One *beta tubulin 6* was targeted by miR1155 in the DS library (Supplementary Table S8). These results suggested that cell plate formation is disturbed by the altered Golgi vesicle transport and phragmoplast formation, and miRNA regulation may play important roles in these processes.

### 3.3. Suppression of Lipid Metabolism Was Responsible for Male Sterility in BS366

Fatty acids and their derivatives are essential components of anther cuticle development and pollen wall formation; lipids are mainly synthesized in the tapetum and transported to the anther wall layers and pollen to promote anther and pollen wall development [39]. The anther cuticle is an extracellular lipidic layer that covers the anther surface [40]. The cuticle is an insoluble and hydrophobic matrix (cutin), which is formed almost exclusively by the interesterification of C16- and C18-polyhydroxy fatty acids [41]. Under cold conditions, the lipid metabolism, oxidation, and transport were repressed in the blue module (Figure 4 and Supplementary Table S2). Genes encoding LTPG6, lipase, and LTP2 involved in lipid transport were among the ten hub genes. Genes involved in lipid metabolism were differentially expressed between cold and control conditions as early as stage 2.2. The sporopollenin biosynthetic process and pollen exine formation were significantly represented. The cutin, suberine, and wax biosynthesis pathway was among the top 20 enriched pathways (Figure 6). The outer wall of pollen and spores, namely the exine, is composed of sporopollenin, which is highly resistant to chemical reagents and enzymes [39]. Sporopollenin, discovered in the outer exine layer of pollen walls, is a lipid- and phenolic-based polymer with high carbon, hydrogen, and oxygen cross-linking Phenylpropanoid pathway derivatives are essential components of sporopollenin in seed plants [42]. Lipid metabolism-related GMS genes involved in the biosynthesis and transport of sporopollenin and anther cutin have been discussed in maize, rice, and Arabidopsis [39]. Male GMS genes such as *OsDPW*, *OsDPW2*, *OsC6*, *AtMs2*, *AtACOS5*, *AtCYP703A2*, and *Zmms44* were previously reported to be involved in the biosynthesis and transport of sporopollenin and anther cutin. In this study, wheat orthologues of *OsDPW*, *AtMs2*, *AtACOS5*, and *AtCYP703A2* were repressed during stages 2.2 and 2.5 under cold conditions. The orthologues of *Zmms44* and *OsDPW2* were induced during stage 3.0 under cold conditions. The expression of a wheat orthologue of *OsC6* was repressed during stages 2.5 and 3.0 under cold conditions. A wheat orthologue of *AtCER3*, a biosynthetic enzyme involved in the production of very long-chain alkanes, was repressed during stage 2.2, 2.5, and 3.0 [43]. It has been reported that glycerol-3-phosphate acyltransferase (GPAT) is mainly involved in anther cutin biosynthesis. Mutation of *AtGPAT6* causes defective tapetum development and leads to the abortion of pollen grains and defective pollen wall formation [44]. Two wheat orthologues of *AtGPAT6* were repressed under cold conditions during stage 3.0. *OsABCG26* was responsible for the transport of cutin to form the anther cuticle [45]. The expression of *TaABCG26*, an orthologue of *OsABCG26*, was repressed under cold conditions. All of these results indicated that the specific repression of lipid- and fatty acid-related processes might impair the cuticle and pollen exine formation in BS366.

### 3.4. The Auxin Signaling Pathway Is Essential to Male Sterility in BS366

The plant hormone auxin plays a critical role in nearly all aspects of plant development including embryogenesis, organogenesis, and reproductive development [46,47]. Auxin regulates transcriptional response via its receptors, TRANSPORT INHIBITOR RESISTANT 1 (TIR1) and AUXIN F-BOX (AFB) [48]. The auxin signaling module is composed of auxin response factors (ARFs), which can either activate or repress the downstream target genes through binding to auxin response elements (AuxRE; TGTCTC/GAGACA) in the promoters [49]. miR160, miR167, and miR393 are involved in auxin signaling through targeting *ARFs*, *AFB*, and *TIR1*, respectively [50]. Studies have shown that auxin regulates anther dehiscence, pollen maturation, and filament elongation during late anther development [51,52]. Plants expressing miR160-resistant *ARF17* showed abnormal stamen structure and reduced fertility [53]. The absence of primexine in the *arf17* mutant leads to pollen wall patterning defects and pollen degradation [54]. This indicates a potential role for *ARF17* in plant fertility. In the degradome data, TraesCS1A02G156600 (*ARF17*-*1A*) was cleaved by miR160 under both conditions, but more cleaved reads were found under control conditions. The cleavage of TraesCS1B02G173700 (*ARF17*-*1B*) and TraesCS1D02G155100 (*ARF17*-*1D*) by miR160 was only found under cold conditions. ARFs interact with auxin/indole acetic acid repressors (Aux/IAAs), which themselves form co-receptor complexes with one of six TIR1/AFB proteins [55]. Two *TIR1* genes (TraesCS1B02G119100 and TraesCS1D02G099900) were cleaved by miR393 under cold conditions. Several Auxin-responsive protein-coding genes, including *IAA12*, *IAA14*, *IAA15*, and *IAA18*, were repressed in BS366 under cold conditions. Auxin-induced protein-coding genes were repressed under cold conditions (Supplementary Table S9). Thus, the cleavage of *TIR1* and *AFR17* may result in male sterility in BS366 through pollenwall pattern formation.

## 4. Materials and Methods

### 4.1. Plant Materials

BS366 is a temperature-sensitive genic male sterile line. It is male sterile at 12 °C (cold) and male fertile at 20 °C (control) with 12 h daylight. Seeds of BS366 were planted in plastic pots in early October. The plastic pots were embedded in the ground in early October 2021 and moved into the greenhouse after natural vernalization. Before the five-leaf stage, ten pots of BS366 plants of uniform growth were selected and then randomly assigned to the cold and control temperature groups. The cold and control treatments of BS366 were carried out according to Liu et al. [56].

### 4.2. Phenotypic Analysis of BS366

Ten BS366 plants were used for the pollen viability evaluation. Anthers from three middle florets of one spike were mixed into three replicates to assess the pollen viability per plant. Anthers were separately crushed and stained with 1% iodine-potassium iodide (I_2_-KI) solution. The fertile pollen ratio of each replicate was calculated for the cold- and control-treated BS366. BS366 plants of uniform growth were selected and then bagged at heading stage. Ten individual plants with three main spikes were chosen for phenotype evaluation. Spikelet seed-setting rate was calculated using (Number of spikelet seeds per ear/Total number of spikelets per ear) × 100% at maturity stage. For microspore phenotype observation, anthers at respective stages under both conditions were collected and fixed in FAA solution (formaldehyde:glacial acetic acid:50% ethanol = 5:5:9). To evaluate pollen viability, cold and control anthers were separately crushed, stained with 1% I_2_-KI solution and photographed under an Olympus BX-53 microscope (Tokyo, Japan). For microspore phenotype observation, the anthers were mashed with tweezers to release the pollen and dyed with improved carbol fuchsin solution. Photographs of microspores and pollen were obtained using an Olympus BX-53 microscope (Tokyo, Japan).

### 4.3. Data Acquisition

The microarray analysis was primarily based on previous Affymetrix microarray data [37]. Samples were collected from different developmental stages of the TGMS line BS366 under different treatment conditions (A, 10 °C with 12 h light/12 h dark; B, 10 °C with 14 h light/10 h dark; E, 20 °C with 12 h light/12 h dark; F, 20 °C with 14 h light/10 h dark). The small RNA and degradome data were downloaded from the NCBI Gene Expression Omnibus (https://www.ncbi.nlm.nih.gov/geo/, accessed on 21 July 2020) with the accession numbers GSE36867 and GSE37134. The sequences of all of the transcripts used to design the probes used in the Affymetrix GeneChip Wheat Genome Arrays (Affymetrix, Santa Clara, CA, USA) were downloaded (https://www.thermofisher.com/, accessed on 21 July 2020) and blasted against the IWGSC reference v1.1 genome with a cut off of 1 × 10^−10^ to convert all of the Array IDs into gene IDs. Only Array IDs that had corresponding genes were retrieved for further analysis.

### 4.4. Bioinformatics Analysis

All array IDs in the Affymetrix GeneChip Wheat Genome Arrays were annotated to the IWGSC reference v1.1 genome, and the array IDs were transformed into gene IDs. For genes with more than one array ID, only the one with a higher average expression level was used for the latter analysis. Differentially expressed genes were analyzed using the limma package in R with a cut-off *p* value less than 0.05 and fold change greater than 2 [57]. After filtering the low-quality reads and removing the adaptor sequences in the small RNA sequencing, clean reads (sRNAs) in the range of 18–30 nt were retained for further analysis. All of the unique sequences were aligned to the IWGSC reference v1.1 genome to obtain tag expression and genomic location information using SOAP [58]. Reads that mapped to wheat rRNA, tRNA, scRNA, snRNA, or snoRNA were removed based on blasting against the National Center for Biotechnology Information (NCBI) (http://www.ncbi.nlm.nih.gov/ accessed on 12 August 2020) and Rfam RNA family databases with default settings. The remaining tags were blasted against the wheat database and then the other plant databases in miRBase release 20 (http://mirbase.org/ accessed on 13 August 2020), using the BLAST search to identify the known miRNAs [59]. Reads that did not annotate to any category were used to predict novel miRNAs using the miRNA prediction program MIREAP (http://sourceforge.net/projects/mireap/, accessed on 15 August 2020). The clean reads for each miRNA were normalized using the following formula: normalized expression (TPM) = mapped read count/total reads × 1000,000. The differentially expressed miRNAs were identified using DEGseq with a threshold of fold change higher than 2 and a *p* value lower than 0.05 [60]. The miRNA targets were predicted using psRobot and TargetFinder software. The Kyoto Encyclopedia of Genes and Genomes (KEGG) and Gene Ontology (GO) analysis was carried out for DEGS in the array data and the target genes of the miRNAs using TBtools [61].

Raw reads of degradome sequencing data were preprocessed to remove adapters and low-quality tags. Clean tags were aligned to the GenBank and Rfam 11.0 databases to annotate the rRNA, tRNA, scRNA, snRNA, and snoRNA, and were mapped to the wheat genome to obtain cDNA sense and antisense tags. The tags mapped to cDNA or mRNA sequences were used to predict the cleavage sites. PAREsnip (http://srna-workbench.cmp.uea.ac.uk/tools/paresnip/, accessed on 28 August 2020) and CleaveLand 3.0 (http://sites.psu.edu/axtell/, accessed on 28 August 2020) were used to identify potentially cleaved targets. All targets were classified into five categories (0, 1, 2, 3, and 4) according to a previous study [62]. Based on the expression characteristics of the wheat transcriptome data, t-plots were built for the high-efficiency analysis of the potential miRNA targets.

### 4.5. Weighted Gene Co-Expression Network Analysis

Gene co-expression networks were constructed using the Weighted Gene Co-Expression Network Analysis (WGCNA) package in R software. The soft-thresholding power β was calculated in the construction of each module using the pickSoftThreshold function of the WGCNA. This method provides a suitable power value for network construction by calculating the scale-free topology fit index for a set of candidate powers, ranging from 1 to 30. In this way, the appropriate power was determined when the index value for the reference dataset exceeded 0.85. Once the soft-thresholding power value was set, the WGCNA algorithms were used to construct the co-expression modules in R software. A one-step network construction method was used to identify the co-expression modules using the blockwiseModules function. The minimum number of genes for each module was set at 40. Network visualization for genes in each module and network between miRNAs and their targets were performed using Cytoscape software version 3.7 [63] with a cut-off of the weight parameter at 0.3.

### 4.6. Sample Preparation, RNA Isolation, and Real-Time qRT–PCR

Anthers of BS366 under different conditions were sampled from three main spikes of fifteen individual plants at the pollen mother cell (PMC) stage, meiosis stage, early uninucleate stage, middle uninucleate stage, and vacuolated stage. All samples were frozen in liquid nitrogen and stored at −80 °C. Total RNA was extracted using TRIzol Reagent (Invitrogen Corp., Carlsbad, CA, USA). The concentration and quality were determined with a Nanodrop spectrophotometer and 1% agarose gel electrophoresis. For real-time qRT–PCR, cDNA was synthesized according to the manufacturer’s instructions (PrimeScript™ RT reagent Kit with gDNA Eraser, Takara; miRcute Plus miRNA qPCR Kit, TIGEN). Differentially expressed genes and miRNAs were validated with a CFX96 Touch™ Real-Time PCR Detection System (Bio-Rad Laboratories, Hercules, CA, USA) using SYBR Green II (Takara). Expression levels of the mRNAs in the samples were normalized against the endogenous wheat actin gene with primer sequences 5′-TACTCCCTCACAACAACCG-3′ and 5′-AGAACCTCCACTGAGAACAA-3′. The expression levels of the miRNAs were normalized using miR-U6 with the primer sequence 5′-GCCTGACACGCACAAATCGAGAAAT-3′. The relative expression levels were calculated using the 2^−ΔΔCt^ method. All the expression analyses were carried out with three technique and biological replicates. Primer sequences were designed using Primer3 input version 4.0.0 (http://primer3.ut.ee/, accessed on 12 June 2021) and primer premier5. The primers for expression validation are listed in Supplementary Table S10.

## 5. Conclusions

In this study, the differential regulation of Golgi vesicle transport and phragmoplast formation processes were responsible for defective cell plate formation in the dyads and tetrads during meiosis. DEGs involved in lipid metabolism, the sporopollenin biosynthetic process, and pollen exine formation may be essential to shrinking microspores at the vacuolated stage. The specific cleavage of *ARF17* and *TIR1* by miR160 and miR393 repressed the auxin signaling pathway during pollen wall pattern formation under cold conditions. The results of our work on these differentially expressed miRNAs and their targets in anthers provide a new understanding of TGMS wheat, which will help us better understand the potential regulatory mechanisms of male sterility in the future.

## Data Availability

The microarray data used for this study are deposited at the National Center for Biotechnology Information Gene Expression Omnibus (https://www.ncbi.nlm.nih.gov/geo/, accessed on 1 June 2022) under accession number GSE200128. The small RNA and degradome data were downloaded from the NCBI Gene Expression Omnibus (https://www.ncbi.nlm.nih.gov/geo/, accessed on 21 July 2020) with the accession numbers GSE36867 and GSE37134.

## Supplementary Materials

The following supporting information can be downloaded at: https://www.mdpi.com/article/10.3390/ijms23158057/s1.
