# Supplementary material for: Integrated Analysis of Microarray, Small RNA, and Degradome Datasets Uncovers the Role of MicroRNAs in Temperature-Sensitive Genic Male Sterility in Wheat
Source: Int J Mol Sci. 2022 Jul 22;23(15):8057. doi: 10.3390/ijms23158057 (PMC9332412; doi:10.3390/ijms23158057)
Supplement: Supplementary file 1 [file ijms-23-08057-s001.zip › ijms-1777671-supplementary.pdf]

Integrated Analysis of Microarray, Small RNA, and Degradome Datasets Uncovers the Role of MicroRNAs in Temperature-Sensitive Genic Male Sterility in Wheat

Yongjie Liu [1,2,†], Dan Li [1,2,†], Shengquan Zhang[1], Liping Zhang[1,2], Jie Gong[1,2], Yanhong Li[1,2], Jiamin Chen[1,2], Fengting Zhang[1], Xiangzheng Liao[1], Zhaobo Chen[1], Yongbo Wang[1], Binshuang Pang[1,2], Jinxiu Ma[1], Xianchao Chen[1], Jiangang Gao[1], Changping Zhao [1,2,*] and Shiqing Gao [1,2,*]

[1] Institute of Hybrid Wheat, Beijing Academy of Agriculture and Forestry Sciences, Beijing 100097, China; liu_yongj@126.com (Y.L.); qdlidan@126.com (D.L.); zsq8200@126.com (S.Z.); lpzhang8@126.com (L.Z.); gjrice@163.com (J.G.); liyanhong201217@163.com (Y.L.); binglinghua66@163.com (J.C.); lyezh@163.com (F.Z.); xiangzhengliao@163.com (X.L.); chzhaobo@126.com (Z.C.); sky999@126.com (Y.W.); pangbinshuang1122@aliyun.com (B.P.); jinxiuma@163.com (J.M.); xcchen10@sina.com (X.C.); gjg86520@126.com (J.G.)
[2] Molecular Genetic Beijing Key Laboratory of Hybrid Wheat, Beijing 100097, China
[3] College of Horticulture, China Agricultural University, Beijing 100193, China
* Correspondence: zhaochangping@baafs.net.cn (C.Z.); gaoshiqing@baafs.net.cn (S.G.)
† These authors contributed equally to this work.

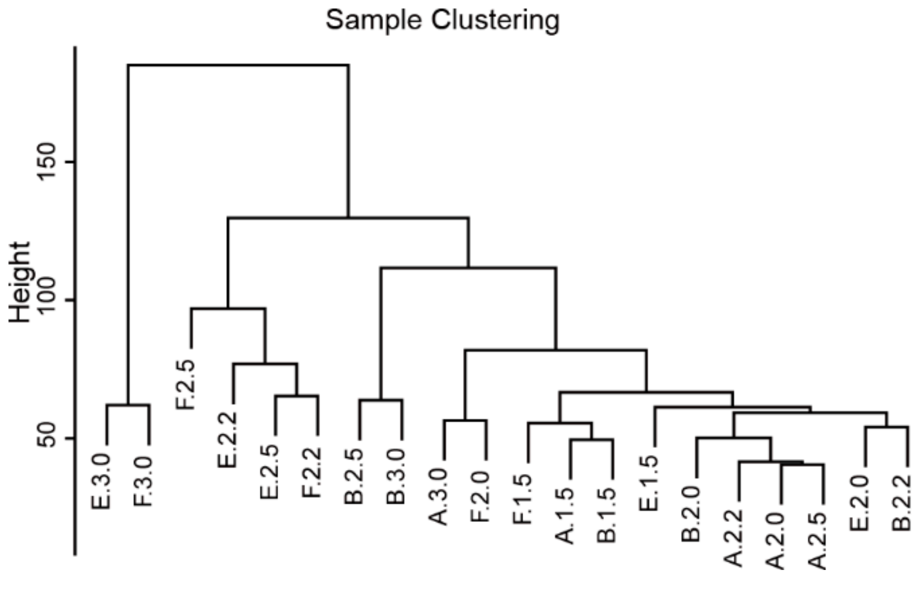

Figure S1 Sample clustering to detect outliers. All the samples were in the cluster. All samples have passed the cuts.

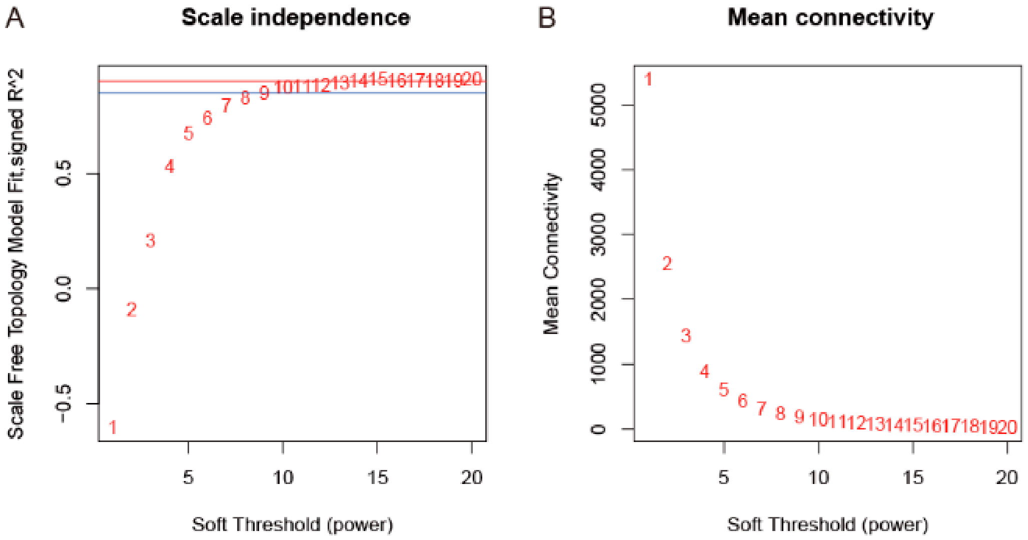

Figure S2 Simulation of soft thresholds and evaluation of weighted network properties for all the expressed genes. (A) The scale-free fit index (y-axis) as a function of the soft-threshold power (x-axis). (B) The mean connectivity (degree, y-axis) as a function of the soft-threshold power (x-axis). Blue line in (A) indicate a scale independence reaches 0.85 and red line indicate 0.90.

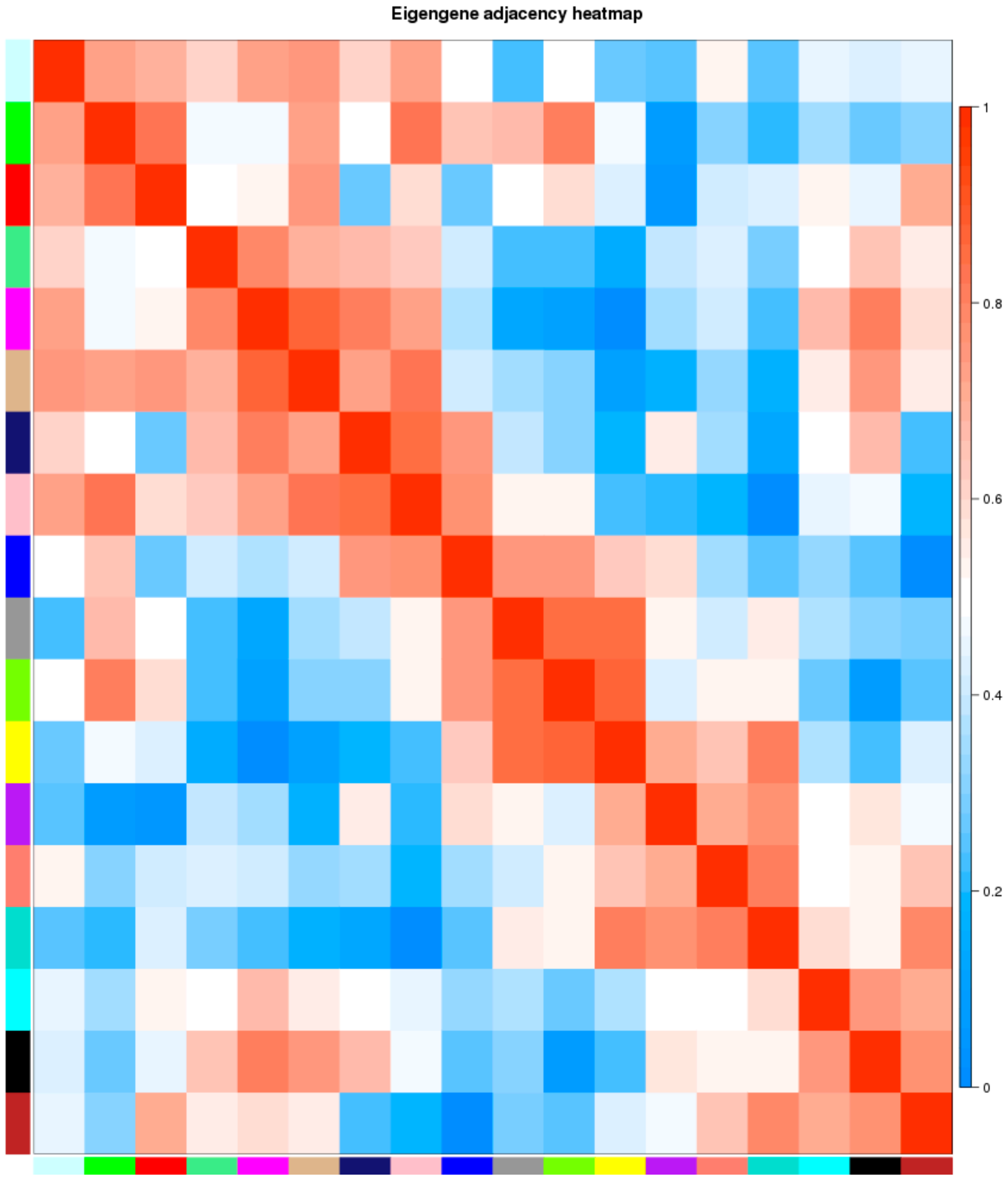

Figure S3 Heatmap plot of the adjacencies modules. Each column and row represent modules with different colors. In the heatmap, red represents positive correlation and blue color indicate negative correlation.

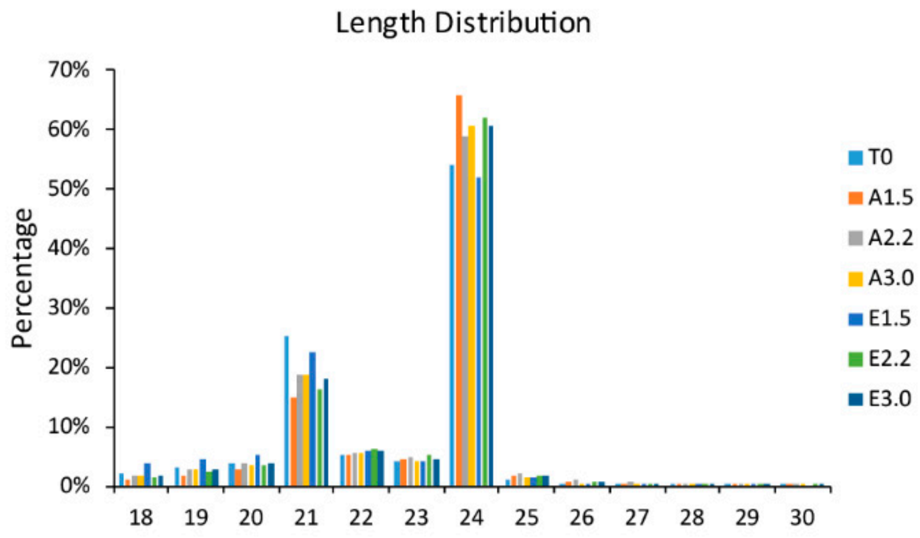

Figure S4 Length distribution of the clean tags in the cold and control conditions. A, cold; E, control.

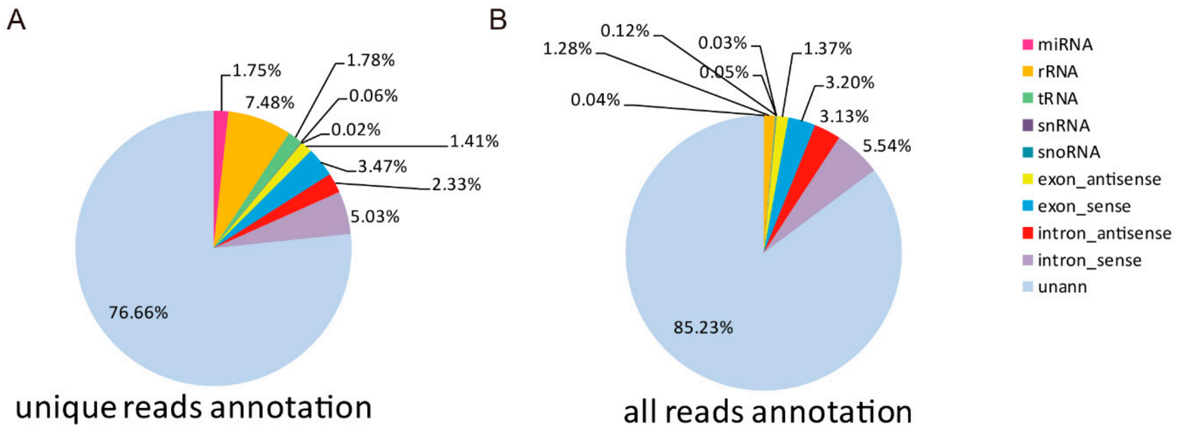

Figure S5 Annotation of the clean tags in all the samples according to the blast to the NCBI and Rfam. The annotation of the unique clean (A) and all (B) tags in all the libraries.

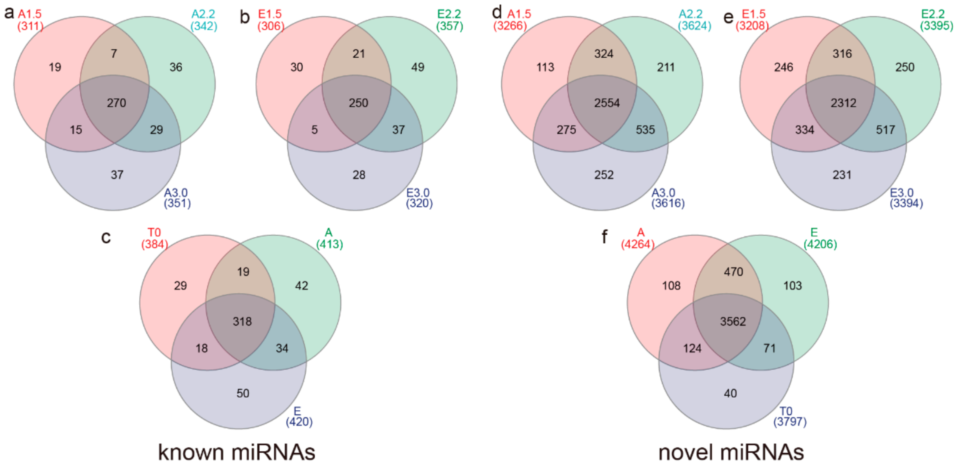

Figure S6 Veen diagram of the expressed known and novel miRNAs. The expressed miRNAs at respective stages in the cold **(a)** and control **(b)** conditions. **(c)** Venn analysis of the expressed miRNAs in the cold, control and T0 stage. The expressed miRNAs at respective stages in the cold **(d)** and control **(e)** conditions. **(f)** Venn analysis of the expressed miRNAs in the cold, control and T0 stage.A, cold; E, control.

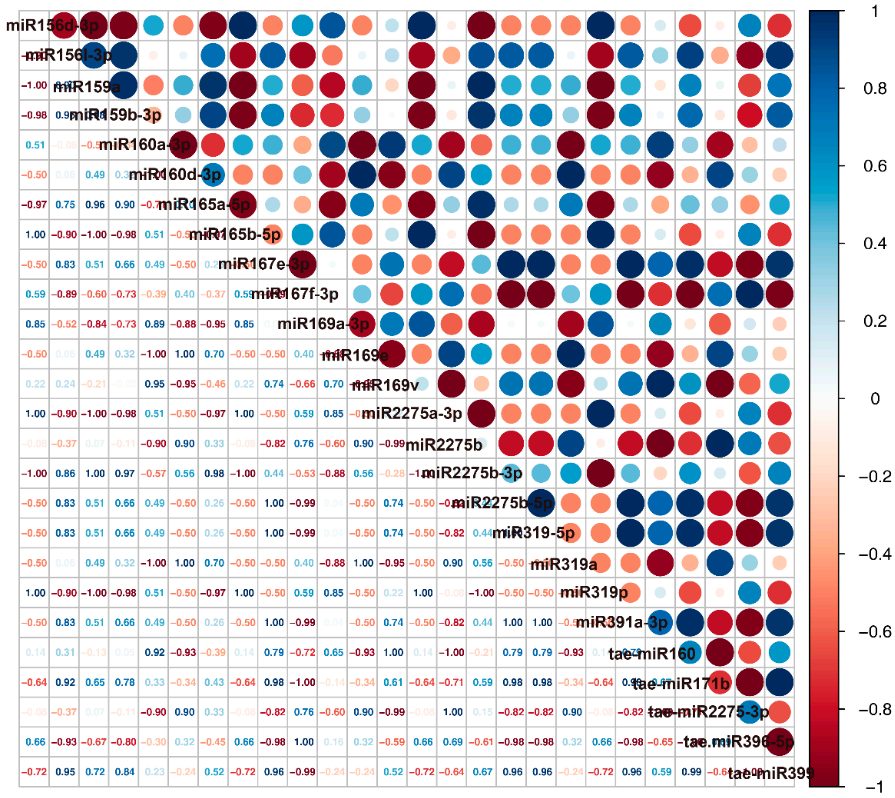

Figure S7 Expressional correlation analysis among conserved known miRNAs. The plots on the diagonal show the names of each miRNA. The values below the diagonal are Pearson correlation coefficients between miRNAs, and the plots above the diagonal are circle plots of compared miRNAs. The size of the circle represents the degree of correlation. Red values and circles indicate a negative correlation; blue values and circles indicate a positive correlation.
